# Supplementary material for: Rapid Removal of Organic Pollutants from Aqueous Systems under Solar Irradiation Using ZrO2/Fe3O4 Nanoparticles
Source: Molecules. 2022 Nov 20;27(22):8060. doi: 10.3390/molecules27228060 (PMC9698733; doi:10.3390/molecules27228060)
Supplement: Supplementary file 1 [file molecules-27-08060-s001.zip › molecules-2029345-supplementary.pdf]

molecules

Supplementary Material

Rapid Removal of Organic Pollutants from Aqueous Systems under Solar Irradiation Using ZrO_2/Fe_3O_4 Nanoparticles

Nemanja Banić [1], Daniela Šojić Merkulov [1], Vesna Despotović [1], Nina Finčur [1], Tamara Ivetić [2], Szabolcs Bognár [1], Dušica Jovanović [1] and Biljana Abramović [1,*]

[1] Department of Chemistry, Biochemistry and Environmental Protection, University of Novi Sad Faculty of Sciences, Trg Dositeja Obradovića 3, 21000 Novi Sad, Serbia

[2] Department of Physics, University of Novi Sad Faculty of Sciences, Trg Dositeja Obradovića 4, 21000 Novi Sad, Serbia

* Correspondence: biljana.abramovic@dh.uns.ac.rs

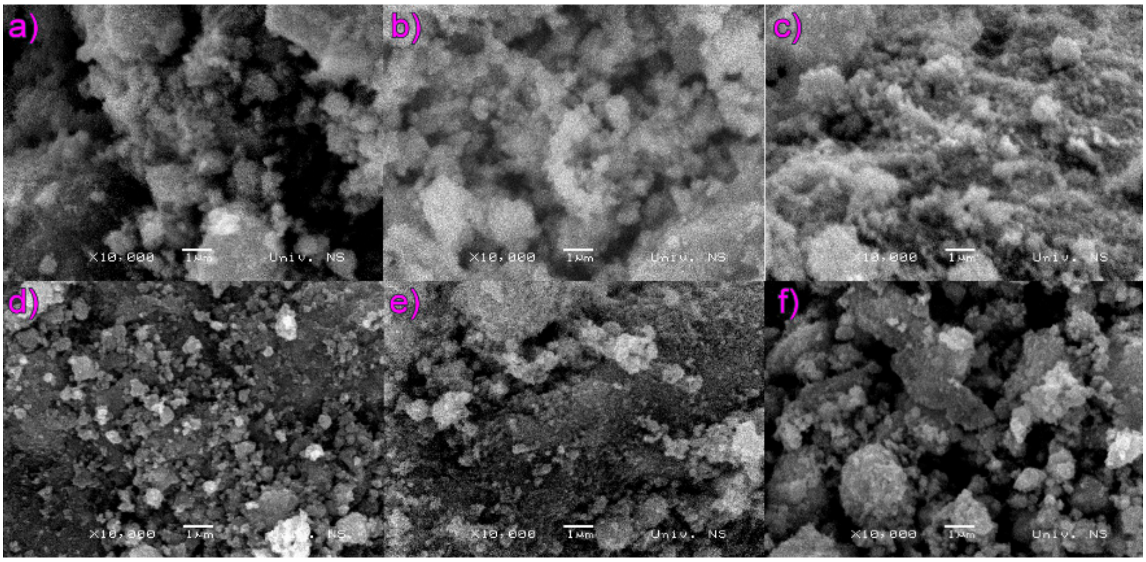

Figure S1. SEM images of particles: (a) Fe_3O_4 and (b) ZrO_2, as well as synthesized nanopowders: (c) $0.9ZrO_2/Fe_3O_4$; (d) $3.5ZrO_2/Fe_3O_4$; (e) $12ZrO_2/Fe_3O_4$ and (f) $19ZrO_2/Fe_3O_4$. Line bar 1 μm.

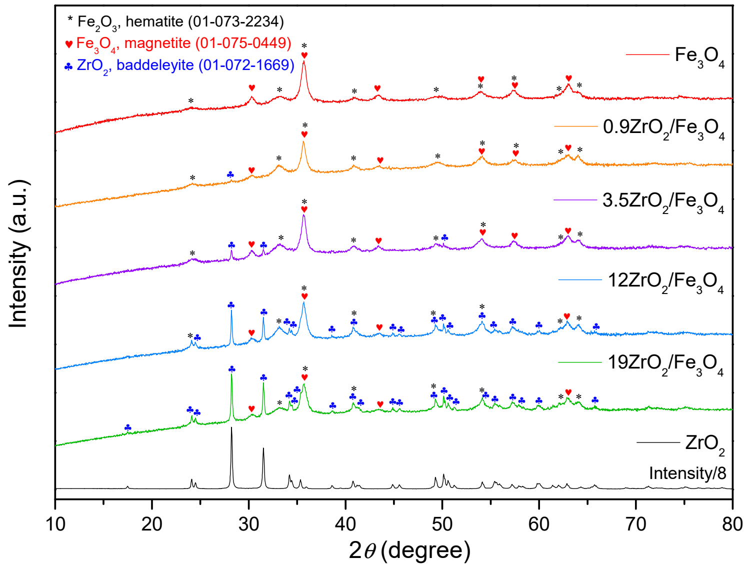

Figure S2. XRD patterns of particles: Fe_3O_4 and ZrO_2, as well as synthesized nanopowders: $0.9ZrO_2/Fe_3O_4$; $3.5ZrO_2/Fe_3O_4$; $12ZrO_2/Fe_3O_4$ and $19ZrO_2/Fe_3O_4$.

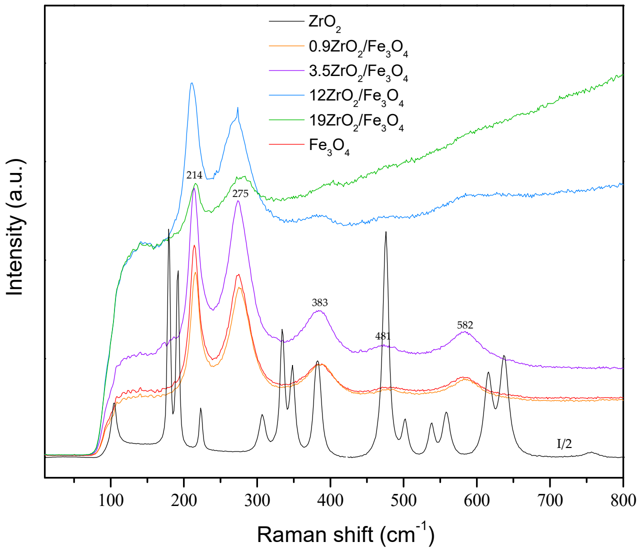

Figure S3. Raman spectra of particles: Fe_3O_4 and ZrO_2, as well as synthesized nanopowders: $0.9ZrO_2/Fe_3O_4$; $3.5ZrO_2/Fe_3O_4$; $12ZrO_2/Fe_3O_4$ and $19ZrO_2/Fe_3O_4$.

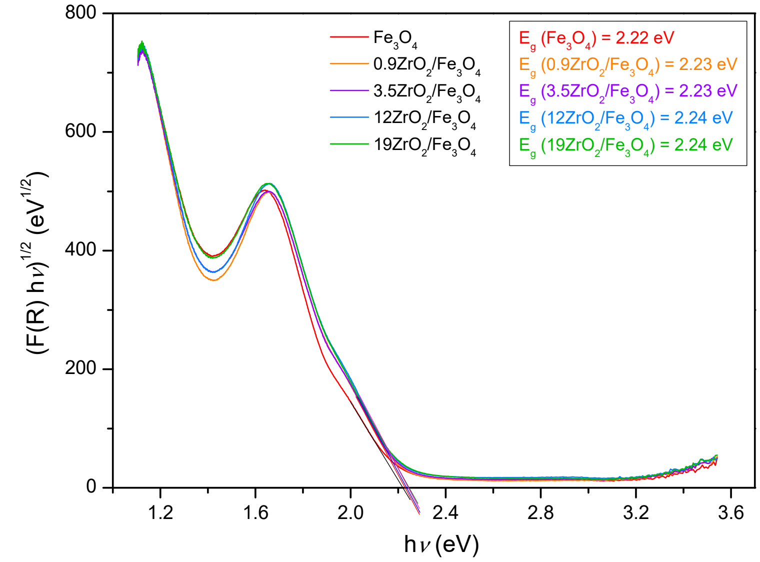

Figure S4. Estimation of particles Fe_3O_4 and synthesized ZrO_2/Fe_3O_4 catalysts optical band energy by $[F(R)\cdot h\nu]^{1/2}$ vs. photon energy (hν) plotting.

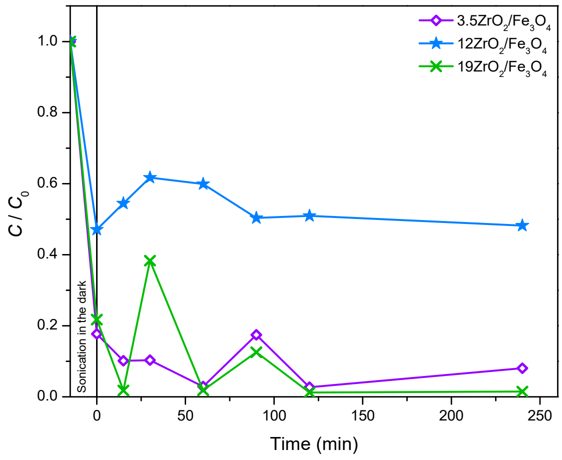

Figure S5. Kinetics of removal of sulcotrione (0.05 mmol/dm^3) at pH 2.8 in the presence of $3.5ZrO_2/Fe_3O_4$, $12ZrO_2/Fe_3O_4$, and $19ZrO_2/Fe_3O_4$ (1.0 mg/cm^3) with 3.0 mmol/dm^3 H_2O_2, under SSI.

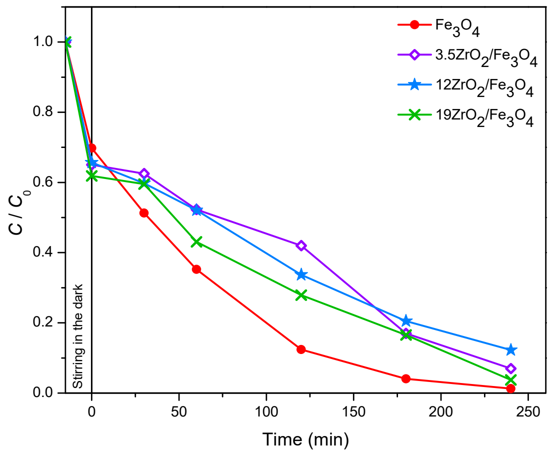

Figure S6. Influence of stirring prior to irradiation on the removal of sulcotrione (0.05 mmol/dm^3) with Fe_3O_4 and various ZrO_2/Fe_3O_4 nanopowders (1.0 mg/cm^3) in the presence of H_2O_2 (3.0 mmol/dm^3) at pH 2.8, using SSI.

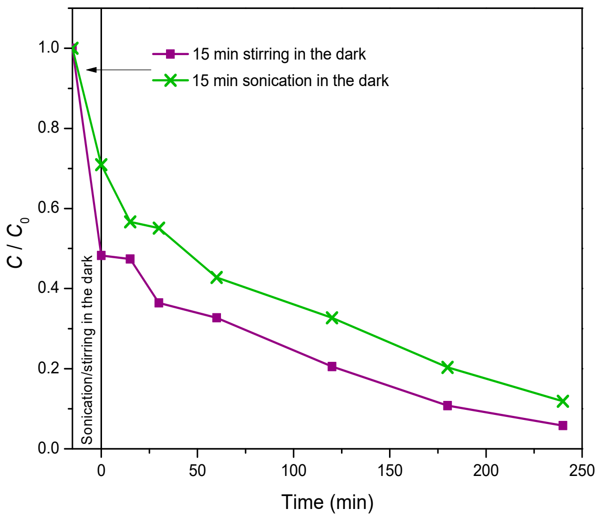

Figure S7. Effect of stirring/sonication on the removal efficiency of fluroxypyr (0.05 mmol/dm^3) in the presence of 45 mmol/dm^3 H_2O_2 and 1.0 mg/cm^3 $19ZrO_2/Fe_3O_4$ at pH 2.8 using solar irradiation.

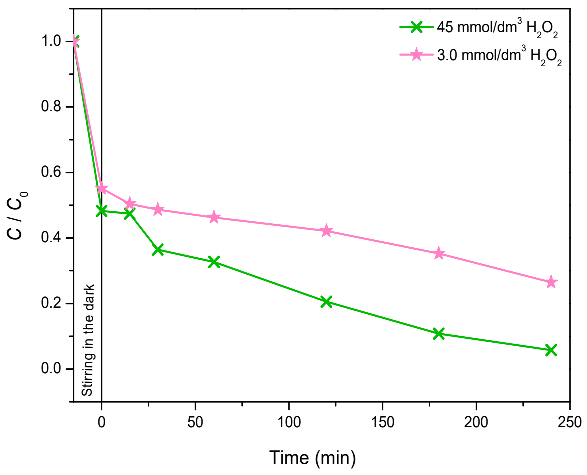

Figure S8. Effect of the initial concentration of H_2O_2 on the removal efficiency of fluroxypyr (0.05 mmol/dm^3) in the presence of 1 mg/cm^3 19ZrO_2/Fe_3O_4 at pH 2.8 using solar irradiation.

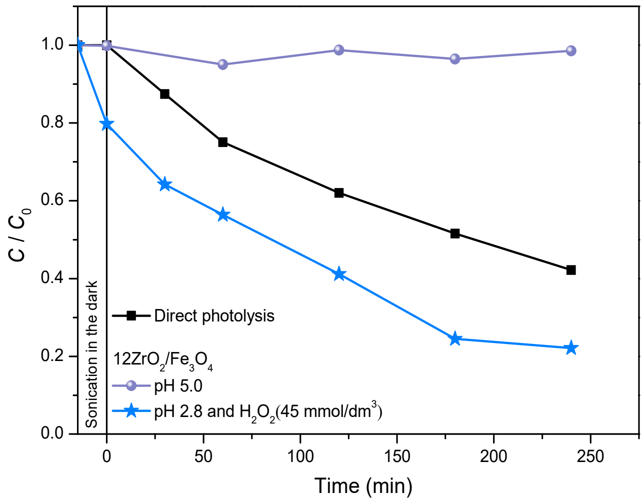

Figure S9. Kinetics of photolytic and photocatalytic degradation of amitriptyline (0.05 mmol/dm^3) in the presence of 12ZrO_2/Fe_3O_4 as a photocatalyst (1.0 mg/cm^3) and using solar irradiation under different experimental conditions.

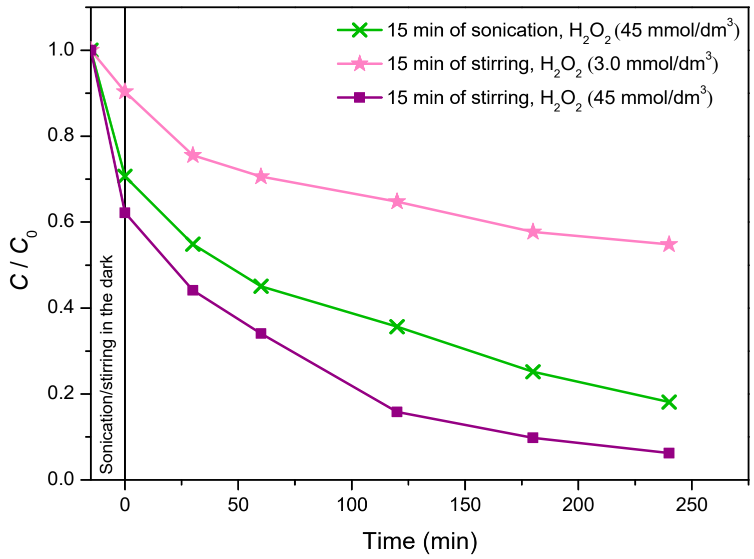

Figure S10. Kinetics of photocatalytic degradation of amitriptyline (0.05 mmol/dm³) in the presence of $19ZrO_2/Fe_3O_4$ (1.0 mg/cm³) as a photocatalyst, H_2O_2 (3.0 and 45 mmol/dm³), at pH 2.8 and using solar irradiation.

Table S1. The physicochemical properties of the analysed natural waters.

Parameter	Natural waters			
	**Danube River**	**Underground**	**Ultrapure**	**Drinking**
pH	7.70	7.62	6.56	7.30
Conductivity at 25 °C (μS/cm)	333	466	4.5	516
TOC (mg/dm^3)	2.30	0.78	<DL	1.80
Fluoride (mg/dm^3)	<DL	0.469	<DL	0.130
Chloride (mg/dm^3)	44.02	61.39	<DL	16.50
Bromide (mg/dm^3)	0.080	0.090	<DL	<0.005
Sulfate (mg/dm^3)	15.52	0.486	<DL	35.0
Nitrate (mg/dm^3)	3.86	0.099	<DL	1.87
Nitrite (mg/dm^3)	2.76	17.53	<DL	<0.01
Calcium (mg/dm^3)	0.136	<DL	<DL	70.49
Potassium (mg/dm^3)	0.030	<DL	<DL	3.75
Lithium (mg/dm^3)	<DL	0.024	<DL	<0.005
Phosphates (mg/dm^3)	0.202	0.052	<DL	<DL
Magnesium (mg/dm^3)	0.078	0.129	<DL	20.3
Sodium (mg/dm^3)	0.043	0.219	<DL	19.2
Ammonium (mg/dm^3)	<DL	15.76	<DL	<0.03
